# Communicative mind-reading in preverbal infants

**DOI:** 10.1038/s41598-018-27804-4

**Published:** 2018-06-22

**Authors:** Tibor Tauzin, György Gergely

**Affiliations:** Cognitive Science Department, Central European University, Budapest, Hungary

## Abstract

Pragmatic theories of communication assume that humans evolved a species-unique inferential capacity to express and recognize intentions via communicative actions. We show that 13-month-old non-verbal infants can interpret the turn-taking exchange of variable tone sequences between unfamiliar agents as indicative of communicative transfer of goal-relevant information from a knowledgeable to a naïve agent pursuing the goal. No such inference of information transfer was drawn by the infants, however, when a) the agents exchanged fully predictable identical signal sequences, which does not enable transmission of new information, or b) when no goal-relevant contextual change was observed that would motivate its communicative transmission. These results demonstrate that young infants can recognize communicative interactions between third-party agents and possess an evolved capacity for communicative mind-reading that enables them to infer what contextually relevant information has been transmitted between the agents even without language.

## Introduction

Humans are a highly social species specially adapted to engage in powerful forms of epistemic cooperation by exploiting their species-unique capacities for communicative information transmission. Through the turn-taking exchange of structured sequences of communicative signals humans transmit a wide range of relevant information across various contexts and knowledge domains. Following Grice’s influential proposals^1^ recent pragmatic theories argue that both verbal and non-verbal communication is an essentially inferential capacity specialized for the expression, recognition, and reconstruction of intentions via communicative actions. This system involves species-unique cognitive adaptations for communicative mind-reading and ostensive-inferential communication that employ dedicated behavioral, inferential, and representational mechanisms^2^. During comprehension such specialized mechanisms support the recipient’s pragmatic inferences to identify the communicator’s intended referent and recover the relevant information he intends to convey about it in the given context. It is argued that even young language learners must rely on context-sensitive pragmatic inferences in the first place to identify and acquire the conventional meanings encoded by novel words on the basis of how competent speakers employ them in various contexts^3,4^.

It has been hypothesized that humans evolved special preparedness to recognize certain intentional actions as ostensive communicative acts: i.e., as actions ostensively performed by an agent with the communicative intention to make manifest relevant information about a referent (his informative intention) for the addressee to infer^3,5^. Within the framework of Natural Pedagogy theory, Csibra and Gergely^6,7^ proposed that preverbal infants possess special sensitivity to ostensive behavioral signals (such as establishing eye-contact or being addressed in motherese). These induce them to attribute communicative intentions to the agent and interpret his subsequent actions as communicative manifestations of his referential and informative intentions. Csibra and Gergely^6,7^ summarized evidence that recognizing ostensive communication induces two types of pragmatic inferences in young infants to (1) identify the intended referent manifested^8^, and (2) recover the relevant information that the communicator intends to convey about the referent (e.g.^9^). More recently, it has been demonstrated that humans’ innate sensitivity to speech sounds also allows young infants to recognize unfamiliar words uttered by an agent as ostensive signals indicating his communicative intention to convey relevant information (e.g.^10,11^). Taken together, these studies provide convergent evidence suggesting that evolved sensitivity to ostensive signals enables even 6- to 12-month-olds to recognize communicative actions that agents produce to manifest their referential and informative intentions for a recipient to infer^12,13^.

Another non-verbal cue that has been hypothesized to induce recognition of ostensive communication is provided by highly contingent turn-taking interactions at a distance between agents^6,14,15^. Human infants show early sensitivity to the conditional probability structure of temporally contingent responses exchanged by interacting agents and react differentially to variable levels of causal dependencies detected in such interactions^16,17^. For example, in a pioneering study, Movellan and Watson^18^ demonstrated that if an unfamiliar robot with no human features had reacted in a highly contingent manner (flashing and beeping) to 10.5-month-old infants’ spontaneous actions, infants gaze-followed the robot’s subsequent change of orientation towards a lateral object. Crucially, when the robot’s responses were not contingent on the infant’s actions (and as such, they were not predictable), no similar gaze-following occurred. This finding, replicated by a number of subsequent studies (e.g.^19,20^), was generally interpreted as showing that detecting highly contingent distal reactivity induces infants to attribute intentional agency to the interactive entity and assign a referential interpretation to its subsequent object-directed orienting response.

In this paper we shall explore an alternative hypothesis proposing that the turn-taking exchange of variable unfamiliar signals functions as an informative cue for infants to identify contingent interactions that may involve communicative transfer of relevant information between agents^5,6,14,15^. As argued by information theory^21^, to serve information transmission the contingently produced signal sequences must contain some degree of unpredictability as exchanging perfectly identical signals could not convey any novel information. Therefore, we predict that turn-taking exchange of fully predictable signal sequences does not provide sufficient evidence for infants to conclude that communicative information transmission has taken place. In contrast, when turn-taking contingent interactions involve exchanging variable (rather than fully predictable) signal sequences, we hypothesize that infants can infer that relevant information may have been transmitted between the communicating agents. As pragmatic theories argue^1,2^, an ostensive communicative action implies that the communicator intends to transmit information that is relevant and new to the recipient. On this ground, we assume that preverbal infants can infer what information has been transmitted only when a relevant event has occurred that would motivate its communicative transfer from a knowledgeable to a naïve agent. Such is the case, for instance, when a situational change is observed by the communicating agent, which would be relevant to the goal pursued by the recipient agent who, however, had not witnessed this event.

## Experiment 1: Turn-taking exchange in the context of goal-relevant situational change

To test this hypothesis, we designed a looking time study in which 13-month-old infants observed turn-taking interactions of unfamiliar animated agents. The agents exchanged variable or identical unfamiliar sound sequences in a context where infants could represent the agents as holding contrary beliefs about the current location of a goal-object that one of them has been pursuing. We examined whether recognizing the communicative exchange of variable signals would lead infants to attribute to the naïve agent a belief about the new location of its goal-object due to the communicative transfer of this relevant information by the knowledgeable agent. The earliest evidence that infants can attribute corrective communicative intentions to agents comes from a recent study with 18-month-olds where one human protagonist provided relevant information verbally to update another agent’s false belief^22^. Experiment 1 employed a similar task to test whether preverbal infants are also capable of attributing informative intentions to a knowledgeable agent and can infer when relevant information has been transmitted to a naïve agent through their turn-taking interactions that involve no verbal communication.

During familiarization, infants saw one unfamiliar entity (with no human features) enter the scene pushing a ball along, stopping next to another such entity already there who remained present throughout the whole event sequence. In the Variable Signals condition the two unfamiliar agents engaged in a turn-taking exchange of variable sequences of melodic tone triplets accompanied by glowing lights emanating always from the sound producing entity. The initial tone in each signal triplet emitted by the first agent was always reproduced exactly as the first item of the second agent’s contingent response triplet. In contrast, the third and/or second tones of the exchanged sound triplets were different and so unpredictable. In the Identical Signals condition the contingent tone sequences produced were always the same and so their exchange involved no unpredictability. In both conditions the first agent then moved on to approach one of two laterally positioned boxes and placed its ball into it. Next, while both agents were present, the ball jumped out of the box and landed in the middle of the scene. In half of the trials, it then proceeded to jump into the other box, while in the other half of the trials, it jumped back into the first box again. The familiarization events always ended by the first agent approaching the box, which at that point contained the ball.

During the test phase, after entering the scene the first agent did not stop to interact with the other agent but went directly to one of the boxes, placed its ball in it, turned around and left the scene. In the presence of the other agent the ball then jumped out of the box and jumped into the other container. After this, the first agent returned and exchanged variable (Variable Signals condition) or fully predictable (Identical Signals condition) tone sequences in a contingent turn-taking manner with the other agent who had been present during the relevant contextual change. It was only then that the returning agent proceeded to approach either the now empty box in which it had originally left its ball or the other box, which currently contained the ball.

**Figure 1:**
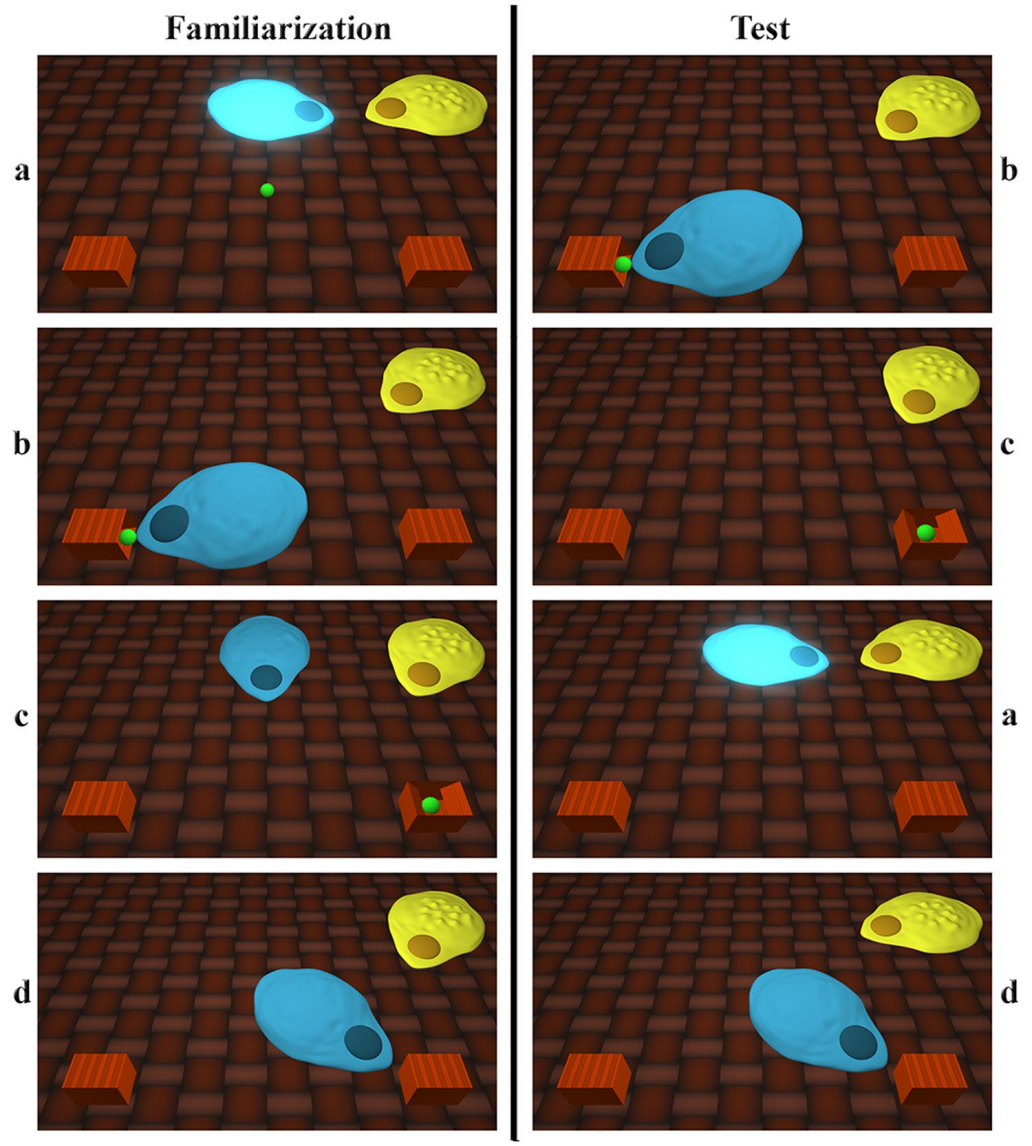
Visual stimuli of the familiarization and test phases of Experiment 1 s. (**a**) turn-taking interaction between the agents, (**b**) hiding of the goal-object, (**c**) change in the location of the goal-object, (**d**) approaching one of the two containers.

We hypothesized that in the Variable Signals condition infants would infer that the turn-taking exchange during the test phase served the communicative transmission of the relevant information about the goal-object’s novel location to the naïve agent. We predicted, therefore, that infants would attribute the returning agent a communication-based belief about the new location of the ball based on the relevant informative content that the knowledgeable agent was inferred to convey during their communicative exchange. Accordingly, infants were hypothesized to expect that after the turn-taking exchange of variable signals the goal-pursuing agent would approach the box, which currently contained the ball. In contrast, the turn-taking interaction in the Identical Signals condition provided no sufficient evidence to sanction the inference that the relevant information about the ball’s current location could have been communicatively transmitted. Therefore, infants had no basis to expect that the naïve agent would approach the currently baited box (Fig. 1).

**Figure 2:**
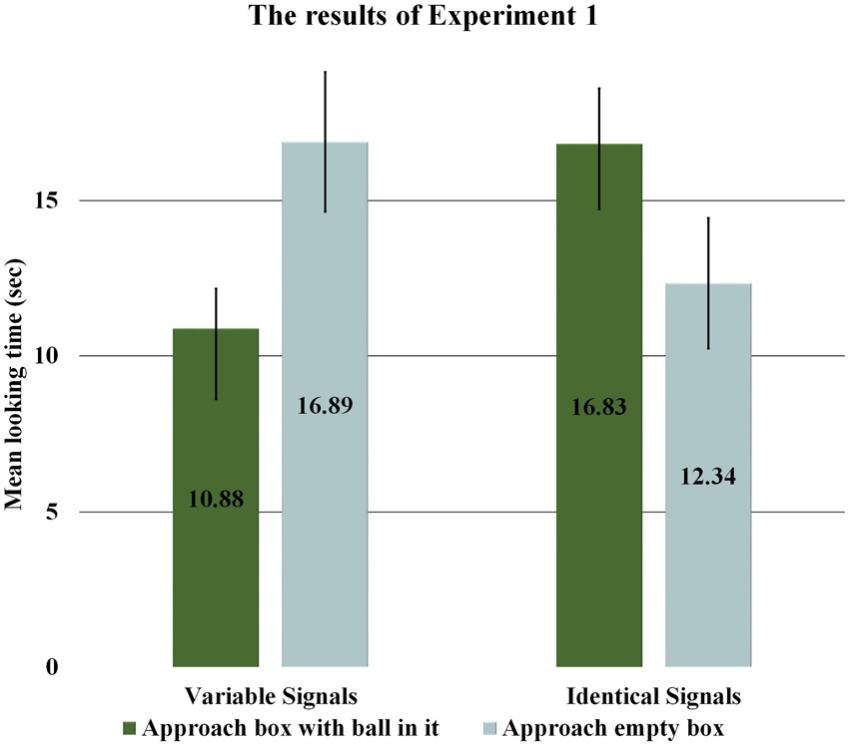
Average looking times (*N* = 40) in Experiment 1. Error bars represent SEM.

To test our hypotheses, we performed robust, semi-parametric GLMM analyses applying a sequential Sidak correction on the log-linked looking time data^23^ with Subject as a random factor. In the Variable Signals condition infants looked significantly longer when the naïve agent went to the now empty box (*M* = 16.89 sec, *SD* = 10.05) to retrieve its ball than when it approached the box which currently contained the ball (*M* = 10.88 sec, *SD* = 5.75; *F*(1, 19) = 8.005, *p* = 0.011). In the Identical Signals condition, however, there was no significant difference in infants’ looking times at the two outcomes (approaching the box with the ball: *M* = 16.83 sec, *SD* = 7.96; approaching the empty box *M* = 12.34 sec, *SD* = 9.4). A comparison of these two conditions revealed a significant Outcome × Condition interaction (*F*(1, 31) = 11.27, *p* = 0.002), while there were no significant main effects of Outcome or Condition (Fig. 2).

These findings support our hypothesis that perceiving turn-taking contingent exchange of variable signal sequences allows 13-month-old infants to infer that relevant contextual information has been communicatively transmitted between the interacting agents. This is indicated by the findings of the Variable Signals condition where infants inferred that, through the communicative exchange, the knowledgeable agent must have conveyed the goal-relevant information about the new location of the goal-object to the naïve agent pursuing the goal. As a result, infants expected the returning agent to approach the box, which currently contained the ball. At the same time, the findings of the Identical Signals condition demonstrate that contingent turn-taking reactivity without variability between the exchanged signals was not sufficient to sanction the inference that communicative transmission of the relevant information had taken place (Fig. 2).

## Experiment 2: Turn-taking exchange in the context of no goal-relevant situational change

Experiment 1 provided evidence that infants can infer that the goal-relevant information has been transmitted to the naive agent by the turn-taking exchange as long as the signals involved variability. In contrast, when the turn-taking interaction consisted of exchanging fully predictable identical signals, infants showed no specific expectation as to which box the returning agent was going to approach following the interactive episode. These findings do not allow to identify what specific belief the goal-pursuing agent may have held about the ball’s location when returning to the scene. According to a belief-tracking account, infants may have represented the returning agent as believing that the goal-object was in the box where it had last placed it, unless this belief had been communicatively corrected. However, this account could not accommodate the results of the Identical Signals condition in which it would predict a belief-based search, but where infants exhibited no specific expectation as to which box the returning agent would approach.

Alternatively, according to an ignorance-based account, since during familiarization the ball repeatedly ended up in either boxes with equal probability, infants may have come to represent the goal-pursuing agent as being ignorant about which one of the two boxes contained the ball when it returned during the test phase. In the Variable Signals condition this state of ignorance could have become updated due to the inferred communicative transmission by the knowledgeable agent of the relevant information about the final location of the goal-object. In contrast, in the Identical Signals condition where this relevant information could not have been communicatively transmitted, the returning agent’s assumed state of ignorance about the ball’s current location should have remained unmodified. Therefore, in the latter condition infants would be predicted to show no specific expectation as to where the ignorant agent would look for its goal-object upon its return. Importantly, the ignorance-based account should predict the same pattern of results to obtain irrespective of whether the goal-object ended up in a new location in the test phase (as in Experiment 1) or if it returned to the same location where the goal-pursuing agent had last placed it. Thus, had the contextual event resulted in no relevant transformation of the goal-object’s original location, the knowledgeable agent could still be expected to communicate this new information about the current location of the ball to the returning agent who was assumed to be ignorant about it. This would predict that infants should expect the returning agent to approach the box currently baited.

Experiment 2 tested this prediction by presenting infants with a contextual change, which resulted in no goal-relevant transformation in the final location of the target object. During the test phase, the ball first jumped out of the box into which the goal-pursuing agent had placed it before leaving the scene, then it jumped back into the same box while the agent was still away. When the agent returned, similarly to Experiment 1, it first engaged in a turn-taking interaction with the other agent, and only then approached one of the two boxes.

**Figure 3:**
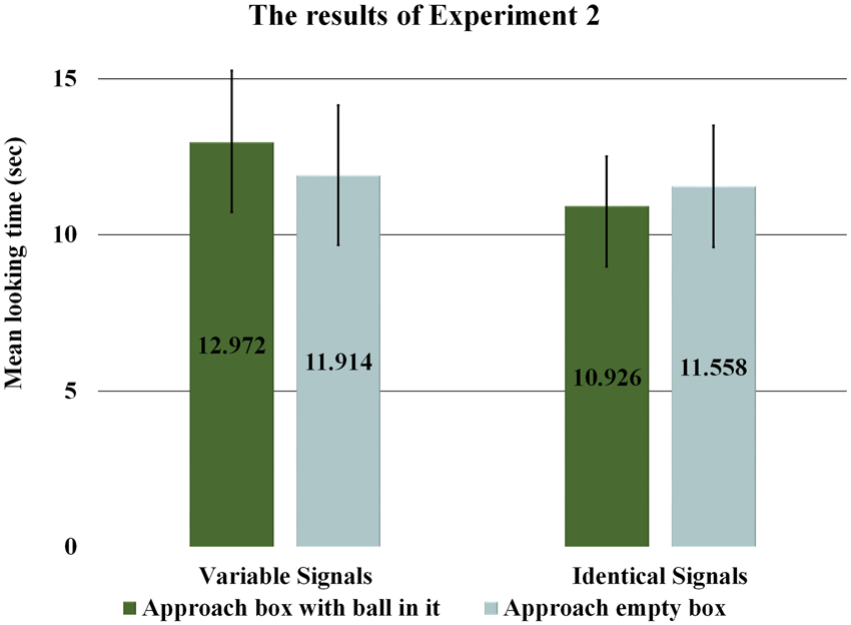
Average looking times (*N* = 40) in Experiment 2. Error bars represent SEM.

The results of Experiment 2 showed no significant difference in infants’ looking times induced by the two outcomes in either of the conditions (Variable Signals – approaching the box containing the ball: *M* = 12.97 sec, *SD* = 10.29, vs. approaching the empty box: *M* = 11.91 sec, *SD* = 10.03; Identical Signals – approaching the box containing the ball: *M* = 10.93 sec, *SD* = 7.12, vs. approaching the empty box: *M* = 11.56, *SD* = 8.7). A 2 × 2 GLMM with Condition and Outcome as main factors also revealed that there were no significant main effects or interaction between these two factors. Importantly, a further GLMM with Experiment as a between-subject factor showed a significant Experiment × Condition × Outcome interaction (*F*(1, 51) = 7.269, *p* = 0.009) without any other significant main effects or interactions. Moreover, when comparing the Variable Signals conditions of Experiment 1 and 2 a further GLMM analysis revealed a significant Outcome × Experiment interaction (*F*(1, 26) = 6.489, *p* = 0.017). The latter results indicate that the pattern of looking times obtained in the Variable Signals condition was significantly different in Experiment 1 as opposed to Experiment 2 (Fig. 3).

In Experiment 2 where no goal-relevant contextual change took place, infants showed no specific expectation as to which box the returning agent would approach in either of the two conditions. Taken alone, the results of the Identical Signals condition would appear to be in line with the ignorance-based hypothesis. However, the findings in the Variable Signals condition cannot be accommodated by this account since the assumed state of ignorance of the returning agent should have been updated by the knowledgeable agent’s communicative information transfer about the location of the goal-object, similarly to Experiment 1. This pattern of results does not seem to support the belief-tracking account either, which assumes that the returning agent held the belief that the goal-object was where it had last placed it, consequently infants should have expected a belief-based search of the goal-object in both conditions.

These findings, therefore, suggest a further alternative interpretation in terms of a relevance-based account that may successfully accommodate all the results reported. This account is based on the assumption that infants will attempt to interpret and relate the turn-taking interactive exchange as providing relevant information about the instrumental goal pursued by the returning agent. When, however, infants find that the social interaction cannot be meaningfully related to the agent’s goal, this interpretive failure may interfere with the maintenance of their expectation that the returning agent is still likely to continue to pursue the originally attributed goal. Consequently, similarly to the Identical Signals condition of Experiment 1 where no communicative transfer of information could be inferred, the non-informative social interaction may lead infants to abandon any specific expectation as to which box the agent is going to approach.

Importantly, the hypothesized relevance-based account relies on a central tenet of pragmatic theories^1,2^, according to which ostensive communication implies that the communicator transmits information that is relevant and new to the recipient. Therefore, in Experiment 2 where infants observed no relevant contextual change that modified the goal-object’s original location, the subsequent turn-taking social exchange could not be interpreted as serving the communicative function of transmitting relevant new information about the position of the goal-object. As a result, the non-informative behavioral interaction that preceded the returning agent’s object approach in Experiment 2 may have also interfered with the infants’ expectation that the agent still maintained and would continue to pursue its original goal. This could account for the finding that following the non-informative intervening social episode infants ceased to show any specific expectation as to which box the agent would be likely to approach.

The relevance-based interpretation is also supported by the significant three-way interaction found between Experiment 1 and 2 and by the significant difference between the Variable Signals conditions of the two experiments. These findings indicate that besides signal variability, registering a goal-relevant situational change that the naïve goal-pursuing agent should know about is also a necessary precondition for infants to infer that the relevant information about the goal-object’s new location has been conveyed by the turn-taking exchange.

## Experiment 3: Belief attribution without turn-taking exchange

One might object, however, that the results of Experiment 1 and 2 are inconsistent with each other. Experiment 1 suggests that in the Variable Signals condition infants inferred that communicative transfer of the relevant information about the ball’s new location has taken place and attributed this communication-based belief to the returning agent. At the same time, Experiment 2 failed to provide evidence that the infants represented the returning agent as holding a perception-based belief about the current location of the ball, although in previous studies infants appeared to be able to represent such beliefs^24,25^.

Crucially, however, it is in line with the relevance-based account that as long as no potentially communicative social exchange preceded the returning agent’s object search during the test phase, infants should be able to maintain the perception-based belief and goal-representation that they had originally attributed to the agent. As a result, in lack of an interference effect infants should expect the returning agent to search for its goal-object in the box where it had last placed it. To test this prediction in Experiment 3 the intervening turn-taking interaction was removed from the test phase, thus the returning agent immediately approached one of the two boxes in search of the ball. In all other aspects, Experiment 3 was identical to Experiment 2.

**Figure 4:**
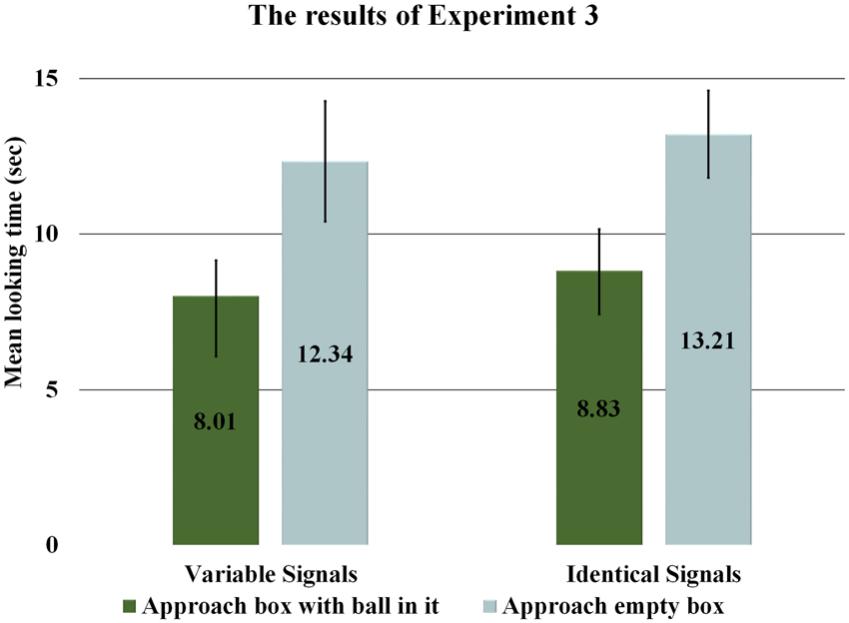
Average looking times (*N* = 40) in Experiment 3. Error bars represent SEM.

In line with the relevance-based account we found that infants in both conditions looked longer when the agent approached the empty box (Variable Signals: *M* = 12.34 sec, *SD* = 8.66; Identical Signals *M* = 13.21 sec, *SD* = 6.28) than when it went to the box, which currently contained the ball (Variable Signals: *M* = 8.01 sec, *SD* = 5.13; Identical Signals *M* = 8.83 sec, *SD* = 5.94). A 2 × 2 GLMM with Condition and Outcome as main factors revealed a significant main effect of Outcome (*F*(1, 16) = 15.121, *p* = 0.001) without any other significant effects. This difference was significant in the Variable Signals (*F*(1, 9) = 7.372, *p* = 0.023) as well as in the Identical Signals condition (*F*(1, 14) = 7.307, *p* = 0.017). A GLMM with Experiment as an additional fixed factor comparing Experiment 2 and 3 showed a significant main effect of Outcome (*F*(1, 40) = 4.50, *p* = 0.04) and a significant interaction between Experiment × Outcome (*F*(1, 40) = 7.281, *p* = 0.01) without any other significant effect. In particular, when looking at the Variable Signals conditions of Experiment 2 and 3 a further GLMM analysis showed a significant Outcome × Experiment interaction (*F*(1, 17) = 6.472, *p* = 0.021) (Fig. 4).

Experiment 3 provided positive evidence that during the test phase infants expected the goal-pursuing agent to approach the container in which it had last placed its ball. This indicates that infants represented the agent as holding a perception-based belief about the location of the goal-object in both conditions of Experiment 3. Moreover, the significant Experiment × Outcome interactions also indicate that a belief-based expectation of goal pursuit was evidenced in Experiment 3, while such an expectation was not maintained in Experiment 2. The latter, we assume, was due to the interference effect induced by the informationally non-relevant social exchange that took place before the returning agent approached of one or the other of the boxes. Thus, in line with the relevance-based account, these findings suggest that infants attributed a perception-based belief to the goal-pursuing agent about the location of its goal-object, unless they observed an informationally non-relevant social interactive episode before the returning agent initiated its object search.

## General Discussion

Our results provide support for the evolutionary-based pragmatic account of ostensive-inferential communication^1–3^ showing that even without language young infants can recognize ostensive communicative acts and infer the relevant information conveyed by communicative agents in a particular context. We demonstrated this in 13-month-old infants who observed two agents engage in a contingent turn-taking exchange of variable and unfamiliar signal sequences, which they recognized as indicative of communicative information transfer. These findings are also in line with previous developmental proposals according to which human infants possess evolved sensitivity to ostensive signals of communication, which induce them to attribute communicative and informative intentions to agents^5,6^.

Apart from providing new empirical support for the evolutionary-based pragmatic account of early communicative competence in humans, the current results also significantly extend our understanding of young infants’ ability to interpret ostensive-inferential communication in several respects. While previous experiments showed that detecting contingent distal reactivity between agents induces referential expectations in infants and trigger referent identification through gaze-following, it has been argued that attributing intentional agency alone is sufficient to account for this effect (e.g.^19^). Such an interpretation, however, cannot accommodate the present results, which are the first to show that turn-taking contingent exchange of variable signals induces a further type of context-based pragmatic inference as well to identify the relevant information that the communicative agent intends to convey about the referent. In a recent study Song *et al*.^22^ showed that relying on verbal communication 18-month-olds can draw both types of context-based pragmatic inferences in question. Our findings demonstrate that this pragmatic inferential capacity for communicative mindreading does not depend on verbal capacities and can be induced by purely non-verbal signals that are indicative of ostensive communication already in 13-month-olds.

The results also demonstrate that the presence of unpredictability in the contingently exchanged signal sequences of a turn-taking interaction is a necessary condition for infants to diagnose that new and relevant information may have been transmitted between communicative agents. As the exchange of fully predictable signals cannot convey new information^21^, the turn-taking contingency in the Identical Signals condition of Experiment 1 and 2 was not sufficient for infants to sanction the inference that relevant information may have been conveyed. Importantly, our results also indicate that signal variability in a turn-taking exchange becomes a crucial factor in contexts where infants expect that information about a relevant event should be transmitted by a knowledgeable and cooperative communicator to a naïve agent.

That non-verbal vocal responses can be communicatively interpreted by infants has also been suggested by recent studies showing that beeping sounds embedded in a turn-taking verbal interaction between humans can subsequently induce categorization and statistical learning effects^26,27^. These intriguing findings leave it unclear, however, whether these effects were due to the beeps being presented as part of a contingent turn-taking exchange, their co-occurrence with speech, or because they were produced by humans. The present study involved neither speech sounds nor human agents, so the communicative interpretation of the unfamiliar non-verbal tone sequences can be unambiguously attributed to the turn-taking contingency structure and the variability of the signal sequences exchanged in a context where a goal-relevant change occurred.

Furthermore, the present study also adds to the currently available evidence on early mind-reading capacities by demonstrating that apart from perception-based beliefs^24,25^, preverbal infants can also attribute communication-based beliefs to other agents as long as they can infer the relevant information that should be transmitted. Such communication-based belief attributions could be induced in 13-month-olds only in a context where they observed a goal-relevant situational change, which they represented as being the relevant new information that should be conveyed to the naïve goal-pursuing agent by a cooperative and knowledgeable communicative partner.

In sum, these results provide support for the pragmatic inferential approach to ostensive communication by demonstrating that even young human infants are capable of communicative mindreading, recognize communicative information transfer based on contingent exchange of variable signals, attribute communicative agency and infer the relevant content of the informative intentions of others without language.

## Methods

### Experiment 1

#### Participants

Forty, healthy, full term infants were assigned randomly either to the Variable Signals (*n* = 20, 11 girls, mean age = 413.85 days, *SD* = 8.79) or to the Identical Signals condition (*n* = 20, 16 girls, 413.2 days, *SD* = 7.71). An additional 11 infants were excluded from the final sample of the Variable Signals condition due to fussiness (4), experimenter error (2), system crash (1) or because the caregiver did not act in line with the instructions (2). Infants who exceeded the maximum looking time in both test trials (2) were also excluded. A further 9 infants were excluded from the final sample of the Identical Signals condition due to fussiness (1), system crash (5) or because exceeding the maximum looking time in both test trials (3).

#### Apparatus

The stimuli were presented using PsyScope X (http://psy.ck.sissa.it) running on a MacPro 4, 1. Videos were displayed on a widescreen (24″) Tobii T-60 XL eye tracker system (Stockholm, Sweden). Looking behavior was recorded both by a video recorder for offline analysis and by Tobii Studio 3.0 running on a Dell Precision T5400.

#### Procedure

Prior to the experiment parents were given a concise description of the study. During the sessions, infants sat on their parents’ lap, approximately 60 centimeters away from the monitor. Parents were asked to wear occluded sunglasses and to hold the infant in the same position, without trying to turn the infants’ torso or head towards the screen if they looked away. Parents were instructed not to speak to or otherwise interact with the baby during the experiment.

#### Stimuli

Each infant watched 4 familiarization and 2 test videos. The videos were 29 and 22.48 seconds long, respectively. In each familiarization video the agents exchanged sequences of sound signals in a turn-taking manner on three consecutive occasions. These interactions were always initiated by the agent who carried a ball along that, after the turn-taking exchanges, it placed into the box on the left side of the screen (from the infant’s perspective). Subsequently, the ball either jumped out and then back into the same box, or jumped into the other box. These two types of familiarization videos were displayed in an ABBA order counterbalancing which of the two videos appeared in initial position across infants. In the test phase the agent hid its ball again in the same box (on the left) before leaving. When returning, it initiated a single turn-taking interaction with the other agent before approaching one of the two boxes. The order of the different outcomes in the test (approaching the box with the ball in it vs. approaching the empty box) was counterbalanced between infants. The counterbalancing of the location visited first (and last) during familiarization and the location approached first during test, thus, yielded four different trial orders appearing with equal frequency across subjects. If the infants looked away during the object-related actions, the videos were temporarily paused until they looked back at the display to ensure that subjects saw all the critical events.

The signal sequences exchanged involved a 50% overlap on average in the Variable Signals condition. For instance, if the first entity produced a sound sequence ‘A-B-C’, the second entity reacted with a signal sequence in such a way that the first element was always repeated, while the second element was repeated in half of the cases and the third element was never repeated (e.g., with ‘A-B-D’ or ‘A-D-E’). The same compositional rule applied also to all the subsequent sequences exchanged. The first sound signal was always different in the 4 familiarization videos. In the Identical Signals condition the signal sequences exchanged were always the same. Therefore, the three elements of the signal triplets emitted by the first entity (for example, ‘A-B-C’) were always reproduced exactly by the second entity (‘A-B-C’). Then the same message (‘A-B-C’) was reproduced again by the first entity, and so on. In the test phase the entities emitted novel tone triplets in the Variable Signals condition, however in the Identical Signals condition they used the same tone triplet as in the familiarization phase.

#### Data analysis

We measured cumulative looking time offline from the end of the test videos. The looking period ended if an infant looked away from the screen for at least 2 seconds or if the looking time exceeded the 30 seconds maximum criterion in a given trial.

### Experiment 2

#### Participants

Forty, healthy, full term infants were assigned randomly either to the Variable Signals (*n* = 20, 12 girls, mean age = 412.2 days, *SD* = 7.98) or to the Identical Signals condition (*n* = 20, 9 girls, mean age = 414.7 days, *SD* = 6.94). We excluded a further 8 infants from the final sample of the Variable Signals condition due to fussiness (2), system crash (4) or because the maximum looking time was exceeded in both test trials (2). From the final sample of the Identical Signals condition an additional 11 infants were excluded due to fussiness (6), system crash (3) or experimenter error (2).

### Experiment 3

#### Participants

Forty, healthy, full term infants were assigned randomly either to the Variable Signals (*n* = 20, 10 girls, mean age = 410.95 days, *SD* = 8.53) or to the Identical Signals condition (*n* = 20, 10 girls, mean age = 406.24 days, *SD* = 8.91). A further 5 infants were excluded from the final sample of the Variable Signals condition because of fussiness (2), system crash (1), experimenter error (1) or because the infant’s looking exceeded the maximum looking time criterion in both test trials (1). From the final sample of the Identical Signals condition an additional 9 infants were excluded because of fussiness (5), system crash (2), because the maximum looking time criterion was exceeded in both test trials (1), or due to experimenter error (1). In the case of one infant in the Identical Signals condition, due to the technical failure of the video recorder, we used the eye tracker data file to measure looking time, as there was no data loss during the test phase.

#### Procedure

Due to the lack of turn-taking interaction in the test, the animations were shorter and lasted for 15.6 seconds.

#### Ethical approval

Our experiments employed only non-invasive procedures for assessing infants’ behavior. Infants were recruited through the Hungarian birth database. All parents were informed about the nature and possible consequences of the study and they signed a consent form about it. We have obtained ethical approval for our studies from the United Ethical Review Committee for Research in Psychology (EPKEB) in Hungary and they were conducted according to the ethical rules and standards regarding psychological experimentation in Hungary.

#### Data availability

The authors declare that all data supporting the findings of this study are available within the paper.

## References

[1] Grice P (1957). Meaning. Philos. Rev..

[2] Sperber, D. & Wilson, D. *Relevance: Communication and Cognition* (Blackwell’s, Oxford, 1986).

[3] Sperber D, Wilson D (2002). Pragmatics, modularity and mind-reading. Mind. Lang..

[4] Vouloumanos, A. & Onishi, K. H. In *Agency and Joint Attention* (eds Metcalfe, J., Terrace, H. S.) 165–177 (OUP, Oxford, 2013).

[5] Gergely G, Jacob P (2012). Reasoning about instrumental and communicative agency in human infancy. Adv. Child Dev. Behav..

[6] Csibra, G. & Gergely, G. In *Processes of Change in Brain and Cognitive Development. Attention and Performance XXI* (eds Munakata, Y., Johnson, M. H.) 249–274 (OUP, Oxford, 2006). 10.1016/j.tics.2005.01.009 15737824

[7] Csibra G, Gergely G (2011). Natural pedagogy as evolutionary adaptation. Philos. T. R. Soc. B.

[8] Senju A, Csibra G (2008). Gaze following in human infants depends on communicative signals. Curr. Biol..

[9] Egyed K, Király I, Gergely G (2013). Communicating shared knowledge in infancy. Psychol. Sci..

[10] Martin A, Onishi KH, Vouloumanos A (2012). Understanding the abstract role of speech in communication at 12 months. Cognition.

[11] Vouloumanos A, Onishi KH, Pogue A (2012). Twelve-month-old infants recognize that speech can communicate unobservable intentions. P. Natl. Acad. Sci. USA.

[12] Harris PL, Lane JD (2014). Infants Understand How Testimony Works. Topoi-Int. Rev. Philos..

[13] Vouloumanos A, Waxman SR (2014). Listen up! Speech is for thinking during infancy. Trends Cogn. Sci..

[14] Csibra G (2010). Recognizing communicative intentions in infancy. Mind Lang..

[15] Deligianni F, Senju A, Gergely G, Csibra G (2011). Automated gaze-contingent objects elicit orientation following in 8-months-old infants. Dev. Psychol..

[16] Watson JS (1972). Smiling, cooing, and “the game”. Merrill Palmer Quart..

[17] Bahrick LE, Watson JS (1985). Detection of intermodal proprioceptive-visual contingency as a potential basis for self-perception in infancy. Dev. Psychol..

[18] Movellan, J. R. & Watson, J. S. The development of gaze following as a Bayesian systems identification problem. *UCSD Machine Perception Laboratory Technical Reports* (2002.01).

[19] Beier JS, Carey S (2014). Contingency is not enough: Social context guides third-party attributions of intentional agency. Dev. Psychol..

[20] Johnson S, Slaughter V, Carey S (1998). Whose gaze will infants follow? The elicitation of gaze-following in 12-month-olds. Developmental Sci..

[21] Shannon CE (1948). A mathematical theory of communication. AT&T Tech. J..

[22] Song H, Onishi KH, Baillargeon R, Fisher C (2008). Can an agent’s false belief be corrected through an appropriate communication? Psychological reasoning in 18.5-month-old infants. Cognition.

[23] Csibra G, Hernik M, Mascaro O, Tatone D, Lengyel M (2016). Statistical treatment of looking time data. Dev. Psychol..

[24] Onishi KH, Baillargeon R (2005). Do 15-Month-Old Infants Understand False Beliefs?. Science.

[25] Kovács ÁM, Téglás E, Endress AD (2010). The social sense: susceptibility to others’ beliefs in human infants and adults. Science.

[26] Ferguson B, Lew-Williams C (2016). Communicative signals promote abstract rule learning by 7-month-old infants. Sci. Rep..

[27] Ferguson B, Waxman SR (2016). What the [beep]? Six-month-olds link novel communicative signals to meaning. Cognition.

